# Hereditary diffuse gastric and lobular breast cancer syndrome associated with germline *CDH1* variants: focus on lobular breast cancer

**DOI:** 10.1007/s00432-025-06222-w

**Published:** 2025-05-14

**Authors:** Giovanni Corso, Francesca Magnoni, Giorgio Bogani, Paolo Veronesi, Viviana Galimberti, Adriana Albini

**Affiliations:** 1 Division of Breast Surgery, European Institute of Oncology, IRCCS, Milan, Italy; 2 Department of Oncology and Hematology, University of Milano, Milan, Italy; 3 Gynecological Oncology Unit, Fondazione IRCCS Istituto Nazionale Dei Tumori, Milan, Italy; 4 European Institute of Oncology, IRCCS, Milan, Italy

**Keywords:** Hereditary lobular breast cancer, *CDH1* gene, Genetic screening, E-cadherin, IGCLC guidelines

## Abstract

**Background:**

Hereditary lobular breast cancer (HLBC) is a distinct subset of hereditary breast cancer primarily associated with germline pathogenic variants in the *CDH1* gene, which encodes E-cadherin, a crucial protein in cell adhesion. Loss of E-cadherin disrupts tissue architecture, contributing to the invasive growth pattern characteristic of lobular carcinoma. *CDH1* mutations are also implicated in hereditary diffuse gastric cancer, predisposing some patients to both cancers. However, variable cancer risk is observed, as many HLBC patients with a family history of gastric cancer do not develop gastric malignancies, reflecting the complex interplay of E-cadherin's role in cell cohesion and tumorigenesis.

**Main body:**

HLBC accounts for 4–5% of lobular breast cancer cases, even in the absence of a personal or family history of gastric cancer. These tumors typically present as hormone receptor-positive (estrogen receptor-positive and progesterone receptor-positive) and are often diagnosed at advanced stages due to their diffuse growth pattern and subtle imaging characteristics. Recent evidence underscores the importance of genetic screening for *CDH1* mutations in women with early-onset bilateral lobular breast cancer or a strong family history of breast cancer. Despite the strong correlation between *CDH1* mutations and HLBC, the absence of diffuse gastric cancer in many patients presents diagnostic challenges. Updated guidelines emphasize targeted surveillance and risk-reduction strategies, including prophylactic mastectomy for high-risk individuals, aiming to improve clinical outcomes.

**Conclusion:**

This mini-review synthesizes recent advancements in understanding the genetics, diagnostic complexities, and clinical management of HLBC. The findings highlight the critical need for early identification and personalized approaches to optimize surveillance and therapeutic strategies for patients with this unique hereditary cancer.

## Background

Hereditary lobular breast cancer (HLBC) is a distinct form of hereditary breast cancer, primarily associated with germline pathogenic variants in the *CDH1* gene, which encodes E-cadherin, a protein crucial for cell adhesion. The loss of E-cadherin disrupts cellular structure, causing the characteristic "single-file" growth pattern in lobular carcinomas (Riedlinger et al. 2021). *CDH1* mutations are also closely linked to hereditary diffuse gastric cancer (HDGC), as loss of E-cadherin compromises cell cohesion across tissues, leading to invasive growth patterns in both diffuse gastric and lobular breast cancers. Interestingly, many women with HLBC have a family history of gastric cancer, though they may not develop gastric cancer themselves, underscoring a dual but variable cancer risk associated with *CDH1* mutations (Riedlinger et al. 2021). HLBC is predominantly associated with lobular breast cancer (LBC), a subtype with specific pathological and radiological characteristics (Corso et al. 2024a). Recent advances in genomic sequencing have identified novel *CDH1* variants, expanding our understanding of the gene’s role in cancer predisposition. Some of these newly identified variants have shown diverse impacts on E-cadherin function, leading to varying degrees of risk for lobular breast cancer and diffuse gastric cancer (Corso et al. 2024b).

The genetic hallmark of HLBC is the *CDH1* gene, and in female carriers, LBC is typically the first phenotype manifestation, even in the absence of diffuse gastric cancer (DGC) (Girardi et al. 2022). Germline *CDH1* pathogenic variants are present in 4–5% of the screened LBC cases, even without a personal or family history of DGC (Corso et al. 2024b). In 2016, Corso et al. identified 14 novel *CDH1* alterations in a study of 482 lobular breast cancer cases, representing approximately 2.9% of cases (Corso et al. 2016). These variants, which contribute to our growing knowledge of HLBC, underscore the need for ongoing refinement of genetic testing protocols. Novel variants, in particular, present challenges in risk interpretation and necessitate careful classification to determine their pathogenicity (Corso et al. 2024b).

HLBC presents unique diagnostic challenges, as its subtle presentation and reduced visibility on imaging make detection difficult (Corso et al. 2024a). Given the association between *CDH1* mutations and both HLBC and HDGC, a family history of gastric cancer should be considered in screening protocols, even if individual patients have not developed gastric cancer themselves. Such an approach may improve early detection and risk assessment for HLBC in high-risk individuals.

HLBC tumors are typically hormone receptor-positive, with around 95% being estrogen receptor-positive and progesterone receptor-positive, and are often accompanied by lobular carcinoma in situ (LCIS) (Gamble et al. 2023).

The loss of E-cadherin in the tumor also makes LBC harder to detect using traditional imaging techniques, leading to later-stage diagnoses. HLBC accounts for 10–15% of invasive breast cancers, with many patients presenting at later stages due to its diffuse growth pattern (Alexander et al. 2022).

Somatic *CDH1* pathogenic variants are associated with invasive lobular carcinoma (ILC), which shows a tenfold higher enrichment than infiltrating ductal carcinoma, underscoring the need for *CDH1* genetic testing in women with ILC (Yadav et al. 2021). Notably, 8% of women with early-onset bilateral lobular carcinoma in situ or ILC carry *CDH1* pathogenic or likely pathogenic (P/LP) variants, even without a family history of DGC, further emphasizing the importance of genetic screening (Petridis et al. 2014).

Furthermore, no co-occurrence of *CDH1* germline variants with *BRCA* mutations was observed, indicating a potential distinct genetic pathway for HLBC (Corso et al. 2024b). This finding has significant implications for genetic screening guidelines, including *CDH1* testing for women with early-onset LBC or those with a family history of breast cancer, regardless of a HDGC history (Girardi et al. 2022). The variability in cancer risk associated with *CDH1* mutations – where some carriers develop gastric cancer, breast cancer, or both – highlights the necessity for a personalized approach to genetic counseling and risk management. Understanding family history across both cancer types enables a more comprehensive risk assessment and more tailored preventive strategies.

In 2018, the Milan Consensus Recommendations and the updated International Gastric Cancer Linkage Consortium (IGCLC) guidelines continued to reflect the growing understanding of genetic risks in HLBC (Corso et al. 2018; Blair et al. 2020).

This mini-review aims to provide a comprehensive overview of the current understanding of HLBC, focusing on key studies and evolving guidelines related to *CDH1* gene variants, which are closely associated with LBC. By synthesizing recent advancements, we aim to highlight areas where further research is needed to optimize patient outcomes in HLBC.

## Key milestones in HLBC research

Key milestones in HLBC research are summarized in Box 1. Between 2013 and 2014, Petridis and Benusiglio enhanced the understanding of HLBC by identifying germline *CDH1* P/LP variants in bilateral LBC cases that did not meet standard IGCLC criteria or had no family history of HDGC. Benusiglio et al. found *CDH1* deleterious variants in three early-onset bilateral LBC cases, which lacked *BRCA1* and *BRCA2* variants and had no family history of DGC. This finding suggested that early-onset LBC might be the first manifestation of HDGC and supported the need for genetic testing in such cases (Benusiglio et al. 2013). Similarly, Petridis et al. identified germline *CDH1* variants in four women with early-onset bilateral LBC, all diagnosed before the age of 50 years, further strengthening the association between *CDH1* variants and LBC, even in the absence of a family history of DGC (Petridis et al. 2014).

In 2016, Corso et al. emphasized the need for updated guidelines to manage germline *CDH1* variants. They identified 14 novel germline *CDH1* alterations in a comprehensive review of 482 LBC cases, reinforcing the importance of early detection and prevention strategies for LBC (Corso et al. 2016).

By 2018–2019, at the Milan meeting chaired by Dr. Giovanni Corso, the first formal clinical criteria for HLBC were established, distinguishing it from HDGC. The experts participating in the meeting recommended *CDH1* genetic testing for individuals with bilateral LBC diagnosed before the age of 50 years and those with unilateral LBC with a family history of breast cancer diagnosed before the age of 45 years. They also provided guidelines for clinical management, including genetic counseling, breast cancer surveillance via MRI, and prophylactic mastectomy for high-risk patients (Corso et al. 2018).

At the 2020 IGCLC meeting, Blair and colleagues confirmed HLBC as a potential independent syndrome, expanding genetic testing to include individuals with early-onset or bilateral LBC, even in the absence of DGC. Guidelines recommended annual breast surveillance starting at the age of 30 years with MRI and included consideration of prophylactic mastectomy for high-risk individuals. The participants in the meeting also noted that individuals with LBC and no family history of DGC might have a lower risk of gastric cancer (Blair et al. 2020).

In 2021, Gamble et al. provided further insights into germline *CDH1* variants but found no specific genotype that could predict whether patients would develop gastric or breast cancer, underscoring the need for individualized clinical management (Gamble et al. 2022).

By 2023, Blair redefined HLBC as a distinct syndrome associated with *CDH1* P/LP variants, which had previously been linked primarily to HDGC. HLBC now includes families with LBC but no known DGC cases, posing a challenge for genetic counseling. The study recommended *CDH1* genetic testing for individuals with a family history of LBC, even in the absence of DGC, and pointed out that other genes, such as *BRCA2*, *CHEK2*, *ATM,* and *PALB2*, are also associated with LBC (Blair et al. 2020).

The increasing focus on LBC was evident at the 2023 San Antonio Breast Cancer Symposium, where funding and research for LBC were key priorities (Lobular Breast Cancer Alliance 2023).

In 2024, Corso et al. reinforced the role of *CDH1* as a key genetic factor in HLBC. Among 394 LBC cases, 1.5% carried *CDH1* P/LP variants. The study also confirmed that 66.7% of tumors in *CDH1* carriers exhibited "second-hit" mechanisms of inactivation, such as loss of heterozygosity, additional somatic variant, or promoter methylation, supporting the role of *CDH1* in LBC development. Furthermore, no co-occurrence of *BRCA1* or *BRCA2* variants was found, suggesting that *CDH1* variants contribute independently to hereditary breast cancer risk. For *CDH1* P/LP variant carriers, risk-reducing surgeries, such as bilateral nipple-sparing mastectomy, are considered alongside psychological counseling to manage the emotional and psychological impact of genetic testing and decision-making related to surgery (Corso et al. 2024b).

Hereditary lobular breast cancer main findings by year

**Table:** 

**2013** Germline *CDH1* variants were identified in three early-onset bilateral LBC cases with no DGC or *BRCA* mutations (Benusiglio et al. 2013)
**2014** Germline *CDH1* variants were identified in four early-onset bilateral LBC cases, all diagnosed before the age of 50 years, without a family history of DGC (Petridis et al. 2014)
**2016** The need for updated guidelines was highlighted after identifying 14 novel *CDH1* alterations in 2.9% of 482 LBC cases (Corso et al. 2016)
**2018** At the Milan meeting, chaired by Dr. Giovanni Corso, clinical criteria for HLBC syndrome, distinguishing it from HDGC, were established, and genetic testing guidelines for HLBC were outlined
**2019** At the Milan meeting, experts continued their efforts by establishing consensus guidelines on genetic counseling, breast cancer surveillance, and prophylactic mastectomy for high-risk HLBC patients
**2020** At the IGCLC meeting, genetic testing criteria for HLBC were expanded, recommending annual breast screening from the age of 30 years. HLBC was redefined as a distinct syndrome from HDGC and linked to *CDH1* P/LP variants. Other genes, such as *BRCA2*, *CHEK2*, *ATM* and *PALB2,* were suggested for testing (Blair et al. 2020)
**2021** *CDH1* genotypes were correlated with HLBC and HDGC, but no genotype predicting cancer type was found, highlighting the need for individualized clinical management (Gamble et al. 2022)
**2024** *CDH1*'s role in HLBC was highlighted, and genetic testing guidelines were updated, emphasizing 'second-hit' inactivation mechanisms that further support *CDH1*'s role in LBC development (Corso et al. 2016)

*DGC* diffuse gastric cancer, *HDGC* Hereditary diffuse gastric cancer, *HLBC* Hereditary lobular breast cancer, *LBC* Lobular breast cancer, *P/LP* pathogenic or likely pathogenic.

## Age of HLBC onset

In *CDH1* P/LP carriers, LBC tends to develop at a significantly younger age, around 42.5 years, than in women with *BRCA1* (48 years) and *BRCA2* (46.5 years) mutations. Petridis et al. identified germline *CDH1* P/LP variants in early-onset bilateral LBC patients diagnosed before age 50, even without a family history of DGC (Petridis et al. 2014). Similarly, Benusiglio et al. reported *CDH1* P/LP variants in bilateral LBC patients, with a mean onset age of around 39 years, even in the absence of a family history of DGC (Benusiglio et al. 2013).

Among women diagnosed with LBC, 1.5% carried *CDH1* P/LP variants, particularly those diagnosed before the age of 45 years or with a positive family history of BC. These carriers developed LBC at a younger mean age, 42.5 years, than those with variants of unknown significance or benign variants, whose mean onset was 51 years (Girardi et al. 2022; Corso et al. 2024b). This earlier onset in *CDH1* P/LP carriers underscores the genetic predisposition in HLBC, where the risk of developing LBC is significantly higher at a younger age than in non-hereditary cases.

## IGCLC guidelines

The IGCLC is a global group of experts involved in researching and managing hereditary gastric cancer, particularly HDGC syndrome. The consortium focuses on the clinical management and genetic testing of individuals with germline *CDH1* variants, predisposing them to both DGC and LBC. Founded in 1999 by international experts, the IGCLC initially narrowed its focus to families with *CDH1* variants because of the significantly increased risk of DGC and, later, LBC. The consortium provides crucial guidance, such as recommending prophylactic total gastrectomy for *CDH1* P/LP variant carriers because of the high lifetime risk of developing DGC. For women with *CDH1* variants, close monitoring for LBC through mammography and MRI is also advised.

The 2020 IGCLC guidelines emphasize the importance of genetic screening for *CDH1* variants in individuals with early-onset or bilateral LBC, even without a family history of HDGC. Looking forward, new guidelines from the 2024 IGCLC consensus conference in Porto are expected to refine genetic screening and risk management strategies for LBC associated with *CDH1* variants. These guidelines will likely focus on personalized surveillance protocols and targeted treatments for high-risk patients.

## Current challenges and future directions

Advances in genetic screening, imaging, and risk-reduction strategies are crucial in managing HLBC. However, significant challenges remain in optimizing these approaches for clinical practice.

Screening young women for *CDH1* mutations, especially those with a family history of LBC or early-onset breast cancer, is a key preventive strategy. Nevertheless, establishing clear criteria for screening in patients without gastric cancer continues to be difficult. Current guidelines recommend testing in cases of early-onset or bilateral LBC, but more refined risk stratification is needed to identify individuals who would benefit most from surveillance or preventive measures (Corso et al. 2016). Additionally, the low frequency of *CDH1* mutations in isolated LBC cases underscores the need for further studies to optimize genetic testing protocols and clarify their implications (Corso et al. 2016).

MRI surpasses conventional techniques, such as mammography, in detecting ILC (Lobbes et al. 2023), particularly for visualizing subtle architectural changes and multifocal or contralateral disease. However, its widespread use is limited by high costs, limited availability, and the potential for tumor size overestimation, leading to unnecessary mastectomies (Alexander et al. 2022; Lobbes et al. 2023). Contrast-enhanced mammography may offer similar accuracy with better accessibility, making it a promising alternative, especially in resource-limited settings (Lobbes et al. 2023).

Bilateral mastectomy is a crucial risk-reduction option for women with *CDH1* P/LP variants because of the high lifetime risk of developing LBC. However, decisions regarding surgery, such as nipple-sparing mastectomy, are complex because of the uncertain penetrance of *CDH1* in breast cancer (Corso et al. 2016). Although guidelines recommend MRI surveillance starting at age 35 for mutation carriers, the role of prophylactic surgery is less defined than its use in *BRCA* mutations (Alexander et al. 2022; Corso et al. 2016). Further research is needed to refine surgery criteria and assess the long-term impact of mastectomy on quality of life and psychological outcomes (Alexander et al. 2022). New CDH1 variants continue to emerge, challenging current classifications of pathogenicity and cancer risk. This variability necessitates advanced functional assays to assess the biological impact of each variant on E-cadherin function (Corso et al. 2024b). Future studies focused on elucidating the penetrance and cancer risk associated with these novel variants will be essential in refining personalized risk management and informing genetic counseling practices for HLBC and HDGC families. In addition, understanding why CDH1 mutations drive tumorigenesis in both gastric and breast tissue is essential for clarifying the variable cancer risks among carriers. Investigating how E-cadherin’s loss uniquely affects these tissues could provide insights crucial to advancing personalized screening and treatment for HLBC and HDGC.

More broadly, several critical questions remain unanswered: What determines the tissue-specific manifestation of CDH1-related cancers—why some carriers develop lobular breast cancer, others diffuse gastric cancer, and some both? How can risk be more accurately stratified among carriers with different variant types and family histories? And how can surveillance and treatment protocols be better adapted to reflect these individual differences? Addressing these gaps will be key to advancing personalized care for HLBC and HDGC families and ensuring appropriate clinical action in the face of evolving genetic information.

## Data Availability

No datasets were generated or analysed during the current study.

## Abbreviations

DGC: Diffuse gastric cancer
HDGC: Hereditary diffuse gastric cancer
HLBC: Hereditary lobular breast cancer
IGCLC: International Gastric Cancer Linkage Consortium
ILC: Invasive lobular carcinoma
LBC: Lobular breast cancer
P/LP: Pathogenic or likely pathogenic
